# A case report of advanced ovarian cancer detected due to an inguinal metastasis in the canal of Nuck

**DOI:** 10.1016/j.ijscr.2019.12.021

**Published:** 2019-12-19

**Authors:** Kazue Togashi, Noriaki Ooyama, Katsuhiko Enomoto, Hirokazu Sato

**Affiliations:** a Department of Obstetrics and Gynecology, Japanese Red Cross Akita Hospital, Akita, Japan; b Department of Pathology, Japanese Red Cross Akita Hospital, Akita, Japan; c Department of Obstetrics and Gynecology, Akita University Hospital, Akita, Japan

**Keywords:** Advanced ovarian cancer, Metastasis model, Hydrocele of the canal of nuck, Inguinal mass, Case report

## Abstract

**Introduction:**

Ovarian cancer has few subjective symptoms, so approximately 40%–50% of cases have already reached stage III or IV by the time of diagnosis. These are advanced stages of the disease and have poor prognosis. Among these cases, less than 3 % are reported to exhibit inguinal lymph node metastasis. This report documents a rare case of advanced ovarian cancer that we detected due to an inguinal metastasis in the canal of Nuck. The work has been reported in line with the SCARE criteria.

**Presentation of Case:**

The patient was a 43-year-old, married, premenopausal woman (G2P1). She was examined by her local practitioner for a chief complaint of a mass in the right inguinal region and was found to have a right inguinal mass. Magnetic resonance imaging (MRI) scans revealed a left ovarian tumor, and she was referred to our department. Rapid intraoperative diagnosis showed a highly atypical serous carcinoma present in both the left ovary and the right inguinal region mass, where the tumor had extended into the canal of Nuck.

**Discussion:**

In this case, the right inguinal mass was ovarian cancer that had metastasized to a cyst in the canal of Nuck via the round ligament of the uterus. Though, many adult women with these types of inguinal hydrocoeles sometimes undergo fine-needle aspiration.

**Conclusion:**

This finding may highlight the need for a careful search for metastasis to the inguinal region in cases of ovarian cancer.

## Highlights

• We present a very rare case of advanced ovarian cancer.
• This is the first report of metastasis to a hydrocele in the canal of Nuck.
• Ovarian cancer can metastasize to a hydrocoele in the canal of Nuck in adult women.

## 1 Introduction

Although the age-adjusted mortality rate for ovarian cancer has declined in recent years, ovarian cancer currently remains the gynecological malignancy that causes the most deaths [1]. With ovarian tumors, there is a relative lack of subjective symptoms during the early stages. Approximately half of all cases are already advanced and in stages III to IV at the time of diagnosis [2]. The majority of current research is focused on the mechanism of ovarian cancer metastasis and dissemination to facilitate early detection of this disease [3]. This report documents an advanced case of ovarian cancer with metastasis to a rare site, so we have included a discussion of the ovarian cancer metastasis mechanism. The work has been reported in line with the SCARE criteria [4].

## 2 Presentation of case

The patient was a 43-year-old woman (G2P1; one spontaneous abortion, one vaginal delivery) with a body mass index of 17.5 kg/m2. She was examined one year earlier by a gynecologist, who detected her uterine myoma. No ovarian mass was observed. In 2016, She noticed a right inguinal mass without any spontaneous pain. She had her gynecologic check-up again. Then she was found to still have multiple uterine myomas, although ultrasound examination revealed an internally heterogenous cyst measuring 32 mm in size in the right inguinal region, as well as an internally heterogenous cystic tumor of the left ovary measuring 60 mm in size. Then she checked blood tests for tumor marker: Carbohydrate antigen (CA) 125 = 139 U/mL, CA 19−9 = 12.4 U/mL, carcinoembryonic antigen (CEA) < 0.5 U/mL.

Her menstrual history included menarche at 12 years of age. She had not reached menopause. Her menstrual cycle was 28 days. She had dysmenorrhea and menorrhagia. Her medical history was unremarkable otherwise. She had no surgical history, allergies, oral medications, or history of alcohol consumption or smoking. Her family history was unremarkable. Her cervical cytology results were negative every year.

**Fig. 1:**
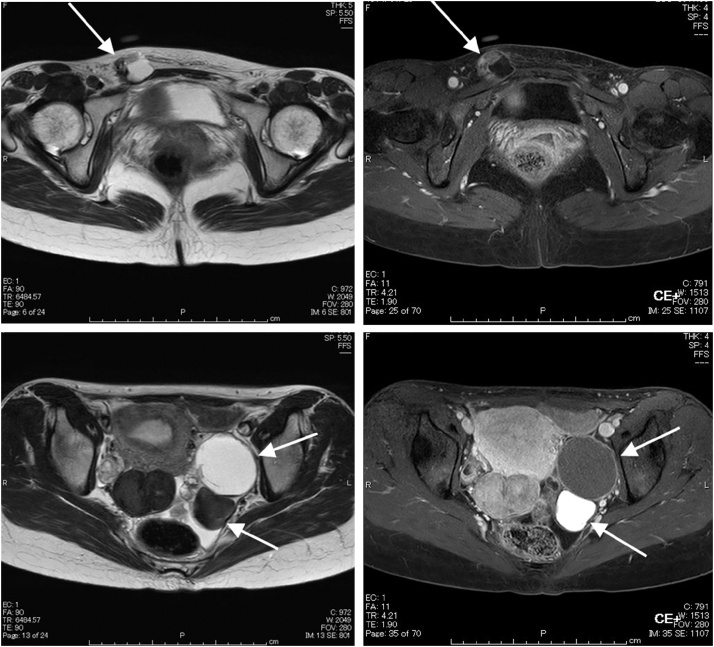
Preoperative MRI scans. Upper left: T2-weighted image, transverse plane. Right inguinal tumor (white arrow) enclosing the serous component; solid components are enclosed by a cystic wall with irregular mural hypertrophy. Upper right: T1-weighted image, fat suppression, transverse plane. Contrast enhancement of the hypertrophic areas of the cystic wall of the right inguinal tumor (white arrow). Lower left: T2-weighted image, transverse plane. Left ovarian tumor visualized as multilocular, with a high-signal intensity ventral chocolate cyst (white arrow, above) and a dorsal solid component (white arrow, below). Lower right: T1-weighted image, fat suppression, transverse plane. Contrast enhancement of the solid components of the dorsal cyst in the left ovarian tumor.

Contrast-enhanced magnetic resonance imaging (MRI) performed 2 weeks after presentation revealed a potentially malignant tumor of the left ovary complicated by an endometrial cyst. The cyst in the right inguinal region was a hydrocoele of the canal of Nuck (Fig. 1).

The patient was referred to our hospital for further examination. Endometrial cytology was performed, and the result was negative. A complete blood count and blood biochemistry were performed, and all values were within the normal ranges. Contrast-enhanced computed tomography (CT) from the neck to the legs revealed no evidence of distant extra-abdominal spread. Gastrointestinal endoscopy was performed as part of a whole-body screening exam, which revealed no abnormalities. The preoperative diagnosis was a left (lt.) ovarian tumor, a right (rt.) inguinal tumor, and multiple uterine myomas. The lt. ovarian tumor appeared to be a borderline malignant or benign tumor.

## 3 Surgical procedure

**Fig. 2:**
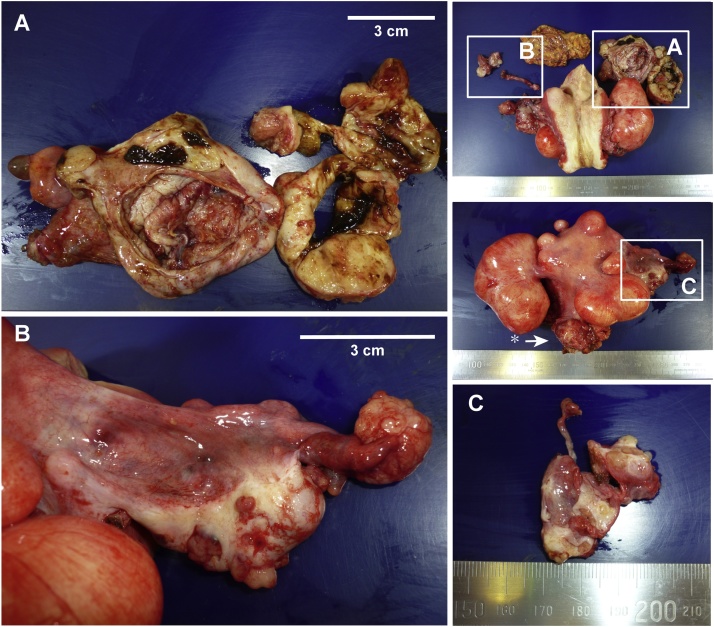
Resected specimen. Right upper (enclosed by the border): Image of the entire resected specimen. Upper image: Ventral. Lower image: Dorsal, pouch of Douglas tumor (*). Disseminated tumors measuring 2 cm in size on the uterosacral ligament are shown; the broad ligament was dissected from the rectum, and the uterus was resected en masse. A) Left adnexa: Chocolate-like cystic fluid and the solid hypertrophic parts of the cystic wall were sent for rapid diagnosis. B) Right adnexa: Disseminated tumors measuring 1 cm in size on the tubal fimbriae. Scattered disseminated tumors measuring 2–3 mm in size on the surface of the right ovary. C) Tumor in the right inguinal region. The cystic lesion contained serous fluid, and we submitted the solid components for rapid diagnosis.

**Fig. 3:**
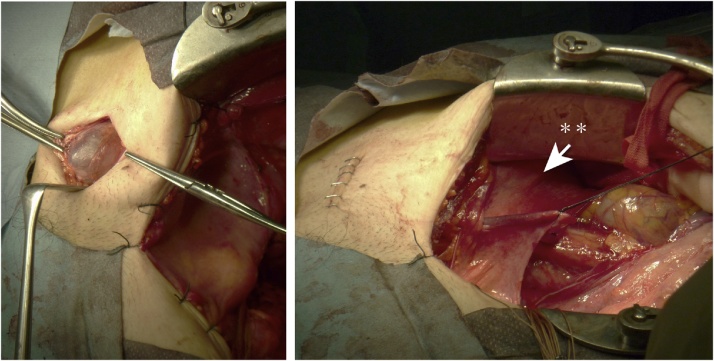
Right inguinal region - intraoperative findings. Left: We transected the external abdominal oblique muscle aponeurosis in the right inguinal region, opened the inguinal canal, and observed a cystic mass to the right and above the pubic symphysis. We observed prolapse of the peritoneum from the peritoneal cavity to the inguinal canal. The cyst was adherent to the round ligament and communicated with the internal inguinal ring, thus resembling a hydrocoele in the canal of Nuck. We dissected the surrounding tissue and performed ligation and transection at the height of the preperitoneal adipose tissue. We then removed the cyst without rupturing it and submitted it for rapid diagnosis. Right: The inguinal canal and the round ligaments (**) from both sides of the abdominal cavity were removed. The internal inguinal ring was not dilated, and no fragility of the posterior wall was noted; therefore, we did not perform herniorrhaphy.

We performed an open total hysterectomy, bilateral adnexectomy, and resection of the rt. inguinal tumor and disseminated tumors. We observed 50 ml of ascitic fluid within the peritoneal cavity, which we submitted for rapid cytological diagnosis. The left ovarian tumor (Fig. 2) was surrounded by adhesions. At dissection, the cystic tumor ruptured. We aspirated the cystic fluid, debulked the tumor, performed resection, and submitted them for rapid histological diagnosis. Disseminated nodules were observed on the serous membrane surface of the rt. ovary and surrounding the rt. tubal fimbriae (Fig. 2B). We also observed disseminated tumors in the pouch of Douglas (Fig. 2 - upper right image, asterisk). We endeavored to resect as many of the disseminated tumors as possible during the hysterectomy. In cooperation with the gastroenterologists, we resected the cystic right inguinal tumor (Fig. 3 - left image) that was continuous with the right round ligament of the uterus (Fig. 3 - right image, two asterisks). We also resected the disseminated tumors on the surface of the rectum and ensured that there were no obvious macroscopic tumors within the peritoneal cavity. No lymph node was palpable. We did not observe a right inguinal hernia or enlargement of the right internal inguinal ring, so we did not perform additional repair. Due to the result of intraoperative pathologic diagnosis, we added omentectomy.

## 4 Pathological diagnosis

**Fig. 4:**
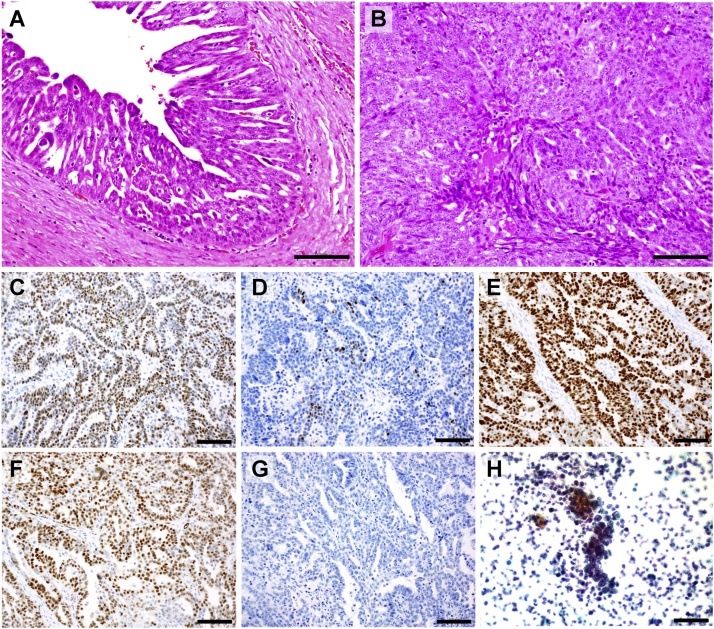
Histopathological findings (print in color). Hematoxylin and eosin (HE) stain: Tumor cells with highly atypical nuclei and prominent nucleoli were observed in the left ovary. The tumor cells were solid and proliferating in a papillary pattern, and we observed several mitotic figures. Similar nuclear atypia was observed in the right inguinal tumor, while some areas exhibited a slit structure and glandular formation, which we believed to represent a similar serous carcinoma (Fig. 4a, b). The scale bars on the bottom right all represent 100 μm. a) Left ovarian tumor, HE 100 × . Tumor cells with highly atypical nuclei and prominent nucleoli were observed. The tumor cells were solid and proliferating in a papillary pattern, and we observed several mitotic figures. b) Right inguinal tumor, HE 100 × . Nuclear atypia similar to the left ovarian tumor was observed, while some areas were observed to exhibit a slit structure and glandular formation. c) Left ovarian tumor, ER 100 × . Weakly positive. d) Left ovarian tumor, PgR 100 × . Slightly positive. e) Left ovarian tumor, p53 100 × . 100 % positive. f) Left ovarian tumor, WT-1 100 × . Positive. g) Left ovarian tumor, PAX8 100 × . Negative. h) Ascitic fluid cytology, 100 × . Nuclear anisocytosis and tumor cells with highly chromatic nuclei that appeared as a bunch of grapes.

Ascitic fluid cytology was positive (Fig. 4h), revealing nuclear anisocytosis and cellular aggregates with abundant chromatin. The intraoperative pathological diagnosis results for the left ovarian tumor revealed serous adenocarcinoma. The right inguinal tumor was also serous adenocarcinoma (Fig. 4).

Hematoxylin and eosin (HE) staining revealed tumor cells with highly atypical nuclei and prominent nucleoli in the left ovary. The tumor cells were solid and proliferating in a papillary pattern, and we observed several mitotic figures. Similar nuclear atypia was observed in the right inguinal tumor, while some areas exhibited a slit structure and glandular formation, which we believed to represent a similar serous carcinoma (Fig. 4a, b).

Immunostaining: p53 = 100 % positive, ER = weekly positive, PgR = slightly positive, PAX8 = negative, WT-1 = positive (Fig. 4c, d, e, f, g).

## 5 Diagnosis

Stage IIIC ovarian carcinoma (FIGO2014)

## 6 Postoperative clinical course

The patient was discharged from the hospital 9 days after surgery. 28days after the surgery, she received six courses of the TC regimen (paclitaxel, carboplatin) every 3 weeks, as well as bevacizumab. The patient has not relapsed for two years.

## 7 Discussion

**Fig. 5:**
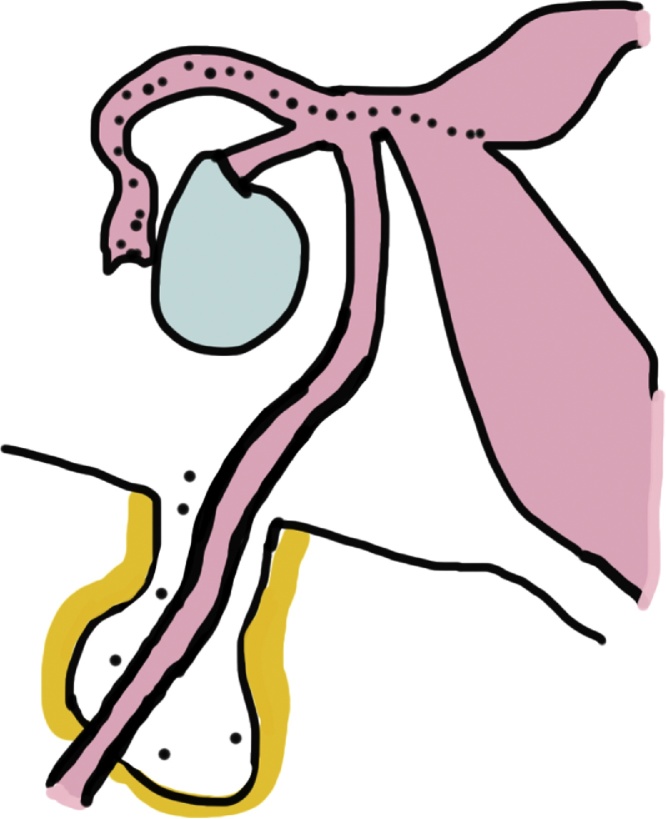
The canal of Nuck.

We encountered a case of ovarian serous carcinoma believed to be a metastasis to the right canal of Nuck. The canal of Nuck is the portion of the processus vaginalis that lies within the inguinal canal in women, and its presence indicates a patent processus vaginalis (Fig. 5). A hydrocoele in the canal of Nuck is thought to occur due to cyst formation in the remnant patent processus vaginalis. Since the canal normally closes after birth, few reports of a hydrocoele in the canal of Nuck in adults are available [5,6]. However, due to the prevalent use of laparoscopic surgery in recent years, scattered case reports describing latent hydrocoeles in the canal of Nuck after intraperitoneal insufflation, even at pressures below 10 mmHg, have emerged [7]. Such hydroceles should be considered to be a possible complication of elevated intraperitoneal pressure.

The relation between endometriosis and hydroceles in the canal of Nuck is interesting. Some Nuck hydrocele cases associated with endometriosis have been reported [[8], [9], [10]]. Notably, the tumor is sometimes closed and has no communication with the abdominal cavity.

Meanwhile, Noguchi et al. reported a case of ectopic pregnancy that occurred in the canal of Nuck in a patient with a closed inguinal ring due to the presence of an endometrial lesion [11]. This case suggests that intraperitoneal cells may migrate into the canal of Nuck when intra-abdominal pressure is increased or endometriosis is present, even if the presence of a patent inguinal ring cannot be clearly confirmed during surgery.

We searched PubMed and Japanese medical abstract databases for cancer cases with a hydrocoele in the canal of Nuck and found four reported cases (Table 1) [[12], [13], [14], [15]]. All reported cases occurred on the right side. Both cancerous lesions and endometriosis were observed in a total of three cases, including our case. Our case was also the only case in which the tumor in the inguinal region was a metastasis of ovarian cancer. Ito et al. reported that they found no endometriosis in the resected uterine specimen, and no communication was noted between the peritoneal cavity and the hydrocoele in the canal of Nuck. Accordingly, the uterine endometrium could not be speculated to have been transplanted into the canal of Nuck and proliferated, and they instead considered that the peritoneum in the processus vaginalis had undergone epithelialization. This process led to the presence of uterine endometrium in the canal of Nuck, which had resulted in endometrioid carcinoma following oncogenesis.

**Table 1:** Cases of malignant tumor of the canal of Nuck reported in adults.

No	Author	Year	Age	Location	Chief complaint	Size	Tissue type	Menopause	Primary lesion	Past history	Outcome
1	Sun et al.	1979	64	R	Painless swelling	8 cm	Low-grade papillary adenocarcinoma	After	Primary	Surgery for cystocele of the urinary bladder	No recurrence survival; 8 years postoperatively
2	Mesko et al.	1988	57	R	N/A	N/A	Clear cell adenocarcinoma	After	Primary	Right herniorrhaphy and endometriosis in the canal of Nuck	Metastasized to the lungs; 2 years postoperatively
3	Hashiguchi et al.	2009	40	R	Painless swelling and skin infiltration	5 cm	Endometrioid adenocarcinoma	Before	Primary	Endometriosis in the right canal of Nuck	N/A
4	Ito et al.	2010	59	R	Painless swelling	4 cm	Endometrioid adenocarcinoma	After	Primary	TAH + BSO for Lt.Ov.Ca. (mucinous cyst adenoma) stage 1a, 14 years earlier	No recurrence survival; 1 year postoperatively
5	Authors	2017	43	R	Painless swelling	3.2 cm	Serous adenocarcinoma	Before	Lt. Ovary	Endometriosis	Ongoing chemotherapy for Lt. Ov. Ca. stage 3c or more

N/A: not available.

T A total of five cases of malignant tumors in the canal of Nuck have been reported, including the present case. All cases occurred on the right side; one case was an adenocarcinoma with low malignant potential, one case was a clear cell adenocarcinoma, and two cases were endometrioid adenocarcinoma; these four cases suggest that the tumors were related to endometriosis in the canal of Nuck. The only case in which the tumor in the canal of Nuck was not the primary tumor is the present case.

We presume that the mechanism of metastasis to a hydrocoele in the canal of Nuck—which is considered to be a closed cavity—is as a result of dissemination based on the cytological and histological evaluations performed in the present case of highly atypical serous carcinoma. Investigating the process of the onset of ectopic endometriosis and ectopic pregnancy may provide new insights into evaluation of ovarian cancer dissemination.

## 8 Conclusion

We encountered an advanced case of ovarian cancer with metastasis to an inguinal cyst that arose in the canal of Nuck, which is a rare site for metastasis to occur. Future studies are required to further clarify the mechanism of ovarian cancer metastasis to the canal of Nuck.

## Sources of funding

There is no source of funding.

## Ethical approval

Our ethics committee approved this report (number; report 1-8). The work has been reported in line with the PROCESS criteria [16].

## Consent

Fully informed written consent has obtained which should be documented in the paper

## Author contribution

Kazue Togashi contributes the paper with writing the paper and data analysis.

Noriaki Ooyama contributes the paper with data collection, data analysis and interpretation.

Katsuhiko Enomoto contributes the paper with data collection, data analysis and interpretation.

Hirokazu Sato contributes the paper with data analysis, interpretation and whole management.

## Registration of research studies

researchregistry5025.

## Guarantor

Noriaki Ooyama is most suitable for the Guarantor.

## Provenance and peer review

Not commissioned, externally peer-reviewed.

## Declaration of Competing Interest

There is no conflict of interest.
